# Perilla Extract improves gastrointestinal discomfort in a randomized placebo controlled double blind human pilot study

**DOI:** 10.1186/1472-6882-14-173

**Published:** 2014-05-27

**Authors:** Sybille Buchwald-Werner, Hajime Fujii, Claudia Reule, Christiane Schoen

**Affiliations:** 1 Vital Solutions GmbH, Hausingerstrasse 6, Langenfeld 40764, Germany; 2 BioTeSys GmbH, Schelztorstrasse 54-56, Esslingen D-73728, Germany; 3 Amino Up Chemical Co., Ltd., 363-32 Shin-ei Kiyota, Saporo, Japan

**Keywords:** Gastrointestinal discomfort, *Perilla frutescens*, Bloating, Abdominal discomfort, Human study, Bowel movement, Passage of gas, Rumbling, Feeling of fullness

## Abstract

**Background:**

Gastrointestinal (GI) discomfort, e.g. bloating or rumbling, is a common symptom in otherwise healthy adults. Approximately 20% of the population, particularly women suffer from gastrointestinal discomfort and this affects quality of life. Recent studies discovered a link between the body and mind, called the gut-brain axis. Psychosocial factors, such as e.g. daily stress may cause altered gut physiology leading to ileum contractions and consequently gastrointestinal symptoms. *In vitro* and *ex vivo* studies clearly showed that a *Perilla frutescens* extract combines prokinetic, antispasmodic and anti-inflammatory effects. The aim of the intervention was to investigate the effects of the proprietary Perilla extract on GI discomfort in healthy subjects with gastrointestinal discomfort and reduced bowel movements in comparison to a placebo product.

**Methods:**

The pilot study was performed according to a double-blind, randomized, placebo-controlled parallel design. Fifty healthy subjects with gastrointestinal discomfort and reduced bowel movements, 30-70 years, documented their GI symptoms, stool frequency and consistency daily during a 2-week run-in phase and a 4-week intervention phase with *Perilla frutescens* extract or placebo. GI symptoms were assessed on a 5-point scale daily and average scores over 14 days intervals were calculated.

**Results:**

All GI symptoms were significantly improved over time by *Perilla frutescens* extract during the intervention phase (bloating: -0.44 ± 0.56, p = 0.0003; passage of gas: -0.30 ± 0.66, p = 0.0264; GI rumbling: -0.55 ± 0.87, p = 0.0014; feeling of fullness: -0.36 ± 0.72, p = 0.0152; abdominal discomfort: -0.54 ± 0.75, p = 0.004), whereas in the placebo group only abdominal discomfort was significantly improved (-0.31 ± 0.55, p = 0.0345). In the subgroup of women results were strengthened and a subscore out of bloating and abdominal discomfort was significantly improved against placebo (95%CI 0.003 to 0.77; p = 0.048).

**Conclusion:**

The demonstrated effects of *Perilla frutescens* extract to improve GI complaints offer very promising results, taking into consideration the challenging set up of a nutritional human study with healthy subjects and in the area of digestive health, which is known for high placebo effects.

**Trial registration number:**

NCT01931930 at ClinicalTrials.gov, Registration date 23^rd^ August 2013.

## Background

Gastrointestinal (GI) discomfort often accompanied by bloating or rumbling, is a common symptom in otherwise healthy adults. Approximately 20% of the population, particularly women, suffers from GI discomfort and these disorders affect quality of life [1-3]. Often, a reduced ability to work and participate in social or recreational activities is reported. Consumer research indicates that today one third of consumers looking for gut health support do not find an effective product that alleviates any of their discomfort [4]. This may be related to the fact that even people with a healthy gut microflora and no food intolerance suffer from digestive discomfort. Recent studies show a link between the body and mind, called the gut-brain axis. Psychosocial factors, such as daily stress may cause altered gut physiology leading to ileum contractions and consequently GI symptoms [5]. In this context, *Perilla frutescens* might be an interesting plant. The proprietary *Perilla frutescens* leaf extract contains a specific ratio of selected flavonoids, particularly vicenin-2 (apigenin-6,8-di-C-glycoside) in combination with rosmarinic acid; all active compounds naturally found in Perilla. *Perilla frutescens* (L.) Britton is an annual edible herbaceous plant native to Asia. Common names are Shiso, Japanese melissea or Japanese basil. Perilla belongs to the mint family, Lamiaceae. The green Perilla leaves are used as tea, food or spice [6]. *In vitro* and *ex vivo* studies clearly showed that the proprietary *Perilla frutescens* extract combines prokinetic and antispasmodic as well as anti-inflammatory effects [7,8]. The study was designed with the aim to develop a health claim substantiated food ingredient and thus comply with EFSA (European Food Safety authority) requirements [9]. It is challenging to show beneficial gut health effects within a target population of healthy people. However, the intention is to demonstrate an improvement of a physiological status which is defined as healthy yet affects quality of life. In addition it is known that human studies investigating GI health show a high placebo effect [10,11]. Digestive health related questionnaires, validated on healthy people are not available to investigate the influence on symptoms and quality of life. EFSA recommends using a validated questionnaire and therefore, it was necessary to evaluate if the questionnaires which were validated on patients presenting with GI disorders (i.e. a diseased population group) would be suitable for application in a study with healthy volunteers with GI discomfort.

## Methods

### Study design

The pilot study was a mono-center, randomized, double-blind, placebo-controlled, human pilot study with parallel design. Prior to trial start, volunteers were screened for their eligibility to take part in the nutritional intervention trial. Eligible subjects were then entered into a 2-week run-in phase to provide baseline measurements of stool frequency and consistency as well as GI discomfort. During the run-in phase subjects did not consume any study products. At the start of the consumption phase (visit 1) the subjects were randomly assigned to one of the two study groups (ratio 1:1). During the 4-week intervention phase the subjects consumed the study product (*Perilla frutescens* extract) or the placebo product (Tapioca starch) according to the randomization scheme. The study was a nutritional study and conducted in accordance with ICH-GCP guideline and in compliance with the declaration of Helsinki. The study was reviewed and approved by the Institutional Review Board (IRB) “Landesärztekammer Baden-Württemberg” and all subjects signed the IRB-approved Informed Consent prior to any procedures being conducted.

### Subject population

**Figure 1:**
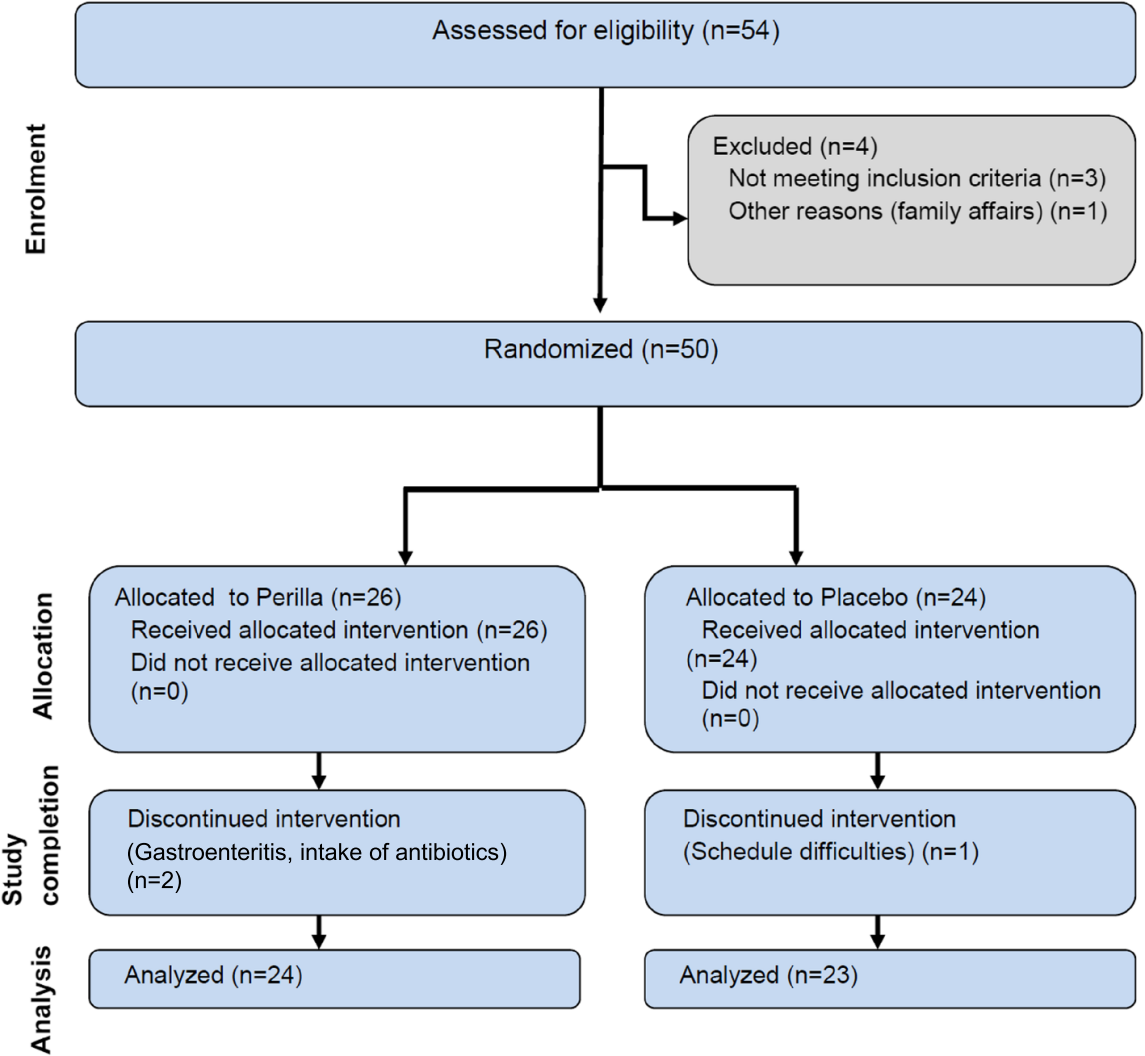
Study flow.

Fifty healthy male and female volunteers between 30 and 70 years of age with reduced bowel movements and gastrointestinal discomfort were enrolled via public notice board and advertisement in newspapers. Forty-seven (9 male and 38 female) volunteers finished the study in accordance to the protocol (Figure 1). The study was performed from July to November 2012 at BioTeSys GmbH, Esslingen, Germany an independent study site which focused on nutritional research. Before study entrance the volunteers were medically checked for their physical condition and they had to fulfill all inclusion criteria and none of the exclusion criteria (Table 1).

**Table 1:** In- and exclusion criteria for the participation at the study

**Inclusion criteria**	**Exclusion criteria**
○ Healthy volunteers without clinical diagnosed diseases with relevant effect on the gastrointestinal system or on visceral motility	○ Subject under prescription for medication for digestive symptoms such as anti-spasmodic, laxatives and anti-diarrheic drugs or other digestive auxiliaries
○ Reduced bowel movements defined as an average of >1 and ≤ 3.5 stools per week for at least the previous 6 months	○ Relevant history, presence of any medical disorder or intake of medication/dietary supplements, potentially interfering with this trial at screening
○ BMI: 19-30 kg/m^2^	○ Subjects with stool frequency of ≤ 1 stool every 7 days or > 3.5 stools per week
○ Gastrointestinal symptoms of at least 5 points	○ Subjects not willing to avoid pre- and probiotics for the duration of the study
○ Male or female	○ Intake of antibiotics in the last 4 weeks and laxatives in the last 2 weeks
○ Age ≥ 30 and ≤ 70 years	○ Change of dietary habits within the 4 weeks prior to screening (for instance start of a diet high in fibers)
○ Nonsmoker	○ Pregnant subject or subject planning to become pregnant during the study; breast-feeding subject
○ Written consent to participate in the study	○ Subjects with history of drug, alcohol or other substances abuse, or other factors limiting their ability to co-operate during the study
○ Able and willing to follow the study protocol procedures	○ Participants anticipating a change in their lifestyle or physical activity levels since this may also influence the results
	○ Known food intolerance or allergy
	○ Subject involved in any clinical or food study within the preceding month

### Intervention

**Figure 2:**
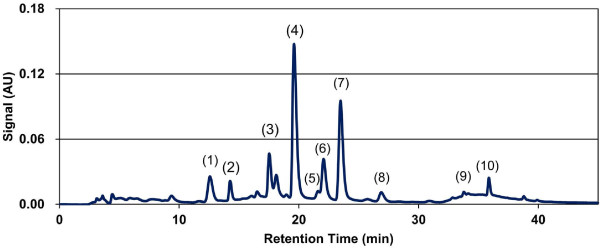
***Perilla frutescens *leaf special extract – HPLC chromatogram.** 1: Caffeic Acid; 2: Vicenin 2 (Apigenin 6,8-di-C-diglucoside); 3: Luteolin 7-O-[β-glucuronosyl(1➔2) β-glucuronide];4: Apigenin 7-O-[β-glucuronosyl(1➔2) β-glucuronide]; 5: Luteolin 7-O- β-glucuronide; 6: Scutellarein 7-O- β-glucuronide; 7: Rosmaric Acid; 8: Apigenin 7-O- β-glucuronide; 9: Luteolin; 10: Apigenin.

The active product tested is a proprietary *Perilla frutescens* extract obtained by water extraction out of dried *Perilla frutescens* leaves, an annual edible herbaceous plant, native to Asia. *Perilla frutescens* is a member of the family Lamiaceae. *Perilla fructescens* extract is a food supplement. The extract is hydrophilic and can easily be dissolved in water. It comprises e.g. 10 different flavonoids, caffeic acid and rosmarinic acid and was characterized by a chromatographic fingerprint in Figure 2 obtained by using the following reverse phase high performance liquid chromatography (RP-HPLC) method. RP-HPLC was performed using a Hitachi apparatus equipped with a L-7420 detector (at 320 nm) and a 250 × 4.6 mm I.D., 5 μm, ODS, Capsule Pack UG-120 (Shiseido, Japan). Separation was achieved with an increasing number of 0.1 percent acetic acid, 5 percent methanol and 10 percent water in acetonitrile (B) in 0.1 percent aqueous acetic acid (A): 0–10 min, 12.5 percent B, isocratic; 10–25 min, 12.5–90 percent B, linear gradient; 25–30 min, 90 percent B, isocratic; 30–31 min, 90–12.5 percent B, linear gradient; and 31–40 min, 12.5 percent B, isocratic at a flow rate of 0.8 ml/min. The method was validated according to ICH-guidelines, using double evaluation and measured against an external reference substances. The proprietary *Perilla frutescens* extract, also known as Benegut®, was supplied by Vital Solutions GmbH, Germany (Dr. Sybille Buchwald-Werner) and the Company Amino Up Chemical Co, Ltd. (Dr. Hajime Fujii), Sapporo, Japan.

The placebo was provided by the manufacturer and matched in size and color to the active product. All subjects were instructed to take 2 capsules daily, one in the morning and one in the evening before food intake with sufficient water for the 4 weeks duration of the intervention phase.

Each capsule contained 150 mg *Perilla frutescens* extract or placebo.

### Assessment

After check for eligibility during screening, subjects were randomly assigned to the study groups after allocating subject numbers in chronologic order by study staff. To ensure double-blind performance the randomization scheme was created by Vital Solutions using the software Randlist with blocksize of 4. Subjects were stratified for gender and age (30-49 years and 50-70 years). All subjects, the investigator and the whole study staff involved in study performance and data analysis were blinded until database lock and deblinding.

After randomization, all subjects documented their GI symptoms, stool frequency and consistency daily during the 2-week run-in phase and the 4-week intervention phase. Additionally questionnaires were filled in at the beginning (visit 1) and at the end of the intervention phase (visit 2). For assessment of GI symptoms, the subjects were asked to grade daily in the evening the average intensity over the previous 24 hours on a 5-point scale from 0 (not at all) to 4 (extremely) for the following GI characteristics: bloating/distension, passage of gas, GI rumbling, feeling of fullness and abdominal discomfort. All answers were evaluated separately and a total score summarizing all assessed GI symptoms as well as a score for bloating and abdominal discomfort was calculated. For evaluation average scores over 14 days intervals were calculated. Additionally, the stool frequency was documented and consistency rated using the Bristol Stool Form Scale [12].

The Patient Assessment of Constipation Symptoms (PAC-SYM) questionnaire, a 12-item self-reporting instrument divided into abdominal, rectal and stool domains, was used to assess the constipation symptoms at the beginning and end of supplementation retrospectively. A scale with 5 categories is used to assess the different symptoms [13]. Additionally, the questionnaire “Patient Assessment of Constipation Quality of Life (PAC-QOL)”, that provides information about the special distraction of daily life and general well-being of volunteers because of constipation, was also assessed [14]. Within the nutritional trial the two sub-scores focused to physical discomfort and satisfaction were recorded. Both questionnaires are develop and validated in a patient population with a history of chronic constipation. The perceived stress questionnaire (PSQ20) [15] is a tool that assesses subjectively experienced stress independent of a specific or objective occasion. It was evaluated before and after the intervention phase and consists of 4 domains, worries, tension, joy and demands with 5 items each. The questionnaire was assessed to include and confirm that the overall stress-levels remained unchanged during the study. Furthermore, subjects were instructed at the beginning of the study not to change their nutrition or sports habits during the study. This was controlled at the end of the study by interview.

### Statistics

Statistics was performed using GraphPad Prism 5.0. The study was conducted as pilot study to get a first overview about efficacy of Perilla extract in the field of gastrointestinal discomfort in humans. Therefore, all efficacy endpoints were evaluated exploratory. As no literature for this specific question was available for the a priori sample size calculation the sample size for the current study was chosen in orientation to other literature in the field of bowel function and gut health [16-20]. Finally, a sample size of 50 volunteers was chosen to be sufficient for overview and basis for further research. Within the analysis, all volunteers with visits under supplementation were statistically evaluated (n = 47). All statistical tests were performed two-sided. A probability level of 0.05 or less was considered to indicate statistical significance. No adjustment to multiple endpoints was performed due to the exploratory character. GI-symptoms were evaluated over 2-week intervals averaging daily scoring data. To show changes over time, a mean score for the run-in period, the first two weeks of intervention and the last two weeks of intervention was calculated. Delta changes of GI symptoms were calculated for the last 2 weeks of intervention phase and baseline levels during run-in phase. Subjects showing improvement of GI symptoms were defined as responders if delta change between the last two weeks of intervention and baseline was <0. Based on this definition, responder rates were calculated for the GI symptoms. As the requirements for a chi square test were not fulfilled due to n < 5 in the group of non-responders in the Perilla group, the results of responder rates were only reported descriptively. Stool consistency was documented each day with bowel movement and evaluated as mean levels in 2-week intervals. Perceived Stress Questionnaire (PSQ20) was assessed before and after supplementation phase. For these previously described parameters, parametric approaches were used, as these data were normally distributed. Paired t-test or Repeated measures analysis of variance (ANOVA) was performed to check for changes over time within group and unpaired t-test was performed for delta changes of Perilla and placebo group to check for differences between groups. Stool frequency, PAC-SYM and PAC-QOL were evaluated with non-parametric tests. Stool frequency (number of days with bowel movement) was evaluated over 2-week intervals and treatment effects were investigated for the delta changes of stool frequency between run-in phase and last 2 weeks of intervention phase. PAC-SYM and PAC-QOL were assessed before and after supplementation phase. Changes within and between groups were checked with Wilcoxon Matched Pairs Signed Rank Test or Mann Whitney Test, as appropriate.

Figures are depicted as scatter plots, indicating mean and 95% confidence interval.

In general, females tend to suffer more from GI discomfort and reduced bowel movements [1,3,21]. Therefore, parameters were additionally evaluated in the subgroup of women.

## Results

Subjects included in the study were in mean 51 years old, with a BMI of 24 kg/m^2^. As shown in Table 2, the study was conducted within a general population with a tendency to reduced bowel movements per week and due to the fact, that women are suffering more from gastrointestinal discomfort [1,3,21], more women than men were included. Characteristics of intervention groups were comparable at baseline (Table 3 run-in).

**Table 2:** Demographic data and baseline characteristics for the whole sample size and the subgroup women

	**Group (N)**	**Age [a]**	**BMI [kg/m**^**2**^**]**	**Chol [mg/dl]**	**Systolic [mmHg]**	**Diastolic [mmHg]**	**Days with stool/ week**
Whole sample size	Placebo (23)	51 ± 8	24.4 ± 3.5	218 ± 45	120 ± 16	77 ± 9	2.83 ± 0.47
	Perilla (24)	51 ± 12	24.4 ± 3.2	231 ± 46	124 ± 14	77 ± 9	2.75 ± 0.47
Subgroup women	Placebo (19)	50 ± 9	24.2 ± 3.7	222 ± 46	118 ± 17	75 ± 9	2.84 ± 0.50
	Perilla (19)	53 ± 11	24.0 ± 3.4	244 ± 42	123 ± 15	77 ± 9	2.76 ± 0.39

**Table 3:** Mean values and standard deviation (SD) for the whole sample of Gastrointestinal symptoms and the delta changes between the run in phase and the intake phase (week 3-4)

**Whole sample size (N = 47)**	**Placebo**				**Perilla**				**Placebo**	**Perilla**	
	**Run In**	**Intake week 1-2**	**Intake week 3-4**	**p value (ANOVA)**	**Run In**	**Intake week 1-2**	**Intake week 3-4**	**p value (ANOVA)**	**Delta week 3-4 to run in**	**Delta week 3-4 to run in**	**Comparison between groups (95% CI)**
Bloating	1.45	1.44	1.2	0.0733	1.42	1.28	0.98	0.0003	-0.25	-0.44	-0.194 to 0.562
SD	0.74	0.72	0.77		0.81	0.64	0.78		0.71	0.56	
Passage of gas	1.47	1.57	1.37	0.2284	1.6	1.5	1.3	0.0264	-0.10	-0.30	-0.177 to 0.577
SD	0.66	0.53	0.62		0.77	0.62	0.74		0.62	0.66	
GI rumbling	1.51	1.33	1.18	0.0635	1.64	1.27	1.09	0.0014	-0.33	-0.55	-0.251 to 0.685
SD	0.86	0.91	0.83		0.85	0.6	0.81		0.71	0.87	
Feeling of fullness	1.08	1.05	0.83	0.0987	1.03	0.84	0.67	0.0152	-0.24	-0.36	-0.288 to 0.515
SD	0.8	0.59	0.62		0.77	0.63	0.63		0.64	0.72	
Abdominal discomfort	1.15	1.06	0.84	0.0345	1.25	0.92	0.71	0.0004	-0.31	-0.54	-0.156 to 0.621
SD	0.93	0.84	0.81		0.86	0.61	0.74		0.55	0.75	

For the comparison within group p values of repeated measures ANOVA and for the comparison between groups the 95% confidence intervals of differences are shown.

**Figure 3:**
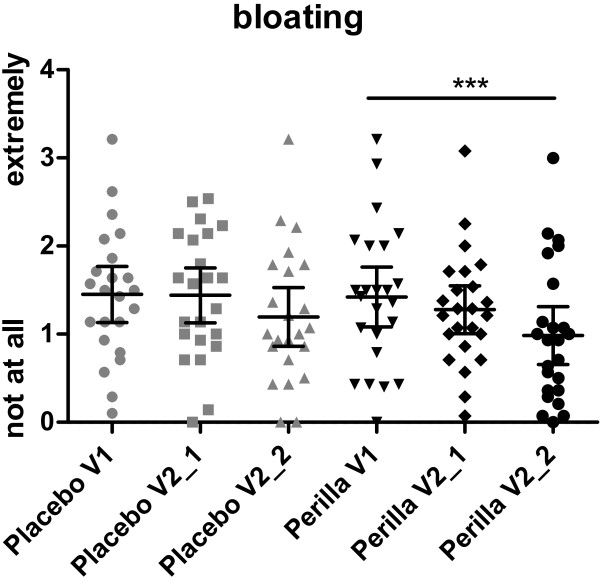
**Distribution of bloating.** Bloating during the run-in period (V1), the first two weeks (V2_1) and the last two weeks (V2_2) of intake phase (Placebo p = 0.0733, Perilla p = 0.0003; Repeated measures ANOVA).

**Figure 4:**
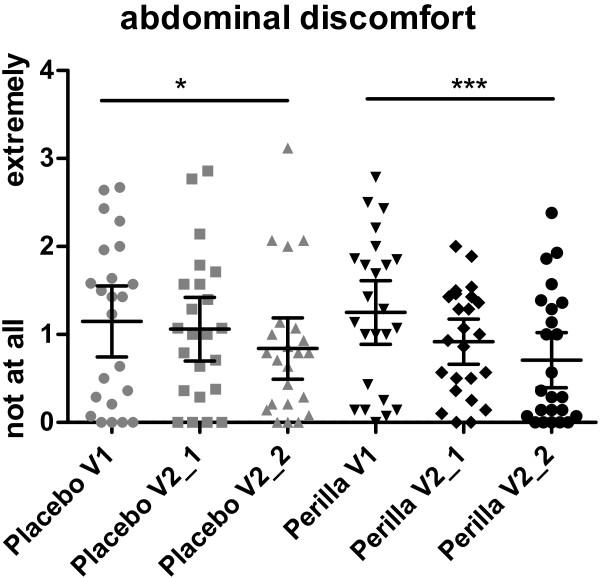
**Distribution of abdominal discomfort.** Abdominal discomfort during the run-in (V1), period, the first two weeks (V2_1) and the last two weeks (V2_2) of intake phase. (Placebo p = 0.0345, Perilla p = 0.0004; Repeated measures ANOVA).

The daily investigated GI discomfort symptoms declined by the intake of Perilla extract. Bloating, passage of gas, GI rumbling, feeling of fullness and abdominal discomfort improved significantly over time (repeated measures ANOVA) from the run-in phase until the end of supplementation phase in the Perilla group (bloating: -0.44 ± 0.56, p = 0.0003; passage of gas: -0.30 ± 0.66, p = 0.0264; GI rumbling: -0.55 ± 0.87, p = 0.0014; feeling of fullness: -0.36 ± 0.72, p = 0.0152; abdominal discomfort: -0.54 ± 0.75, p = 0.004), whereas in the placebo group only abdominal discomfort was significantly improved (-0.31 ± 0.55, p = 0.0345) (Table 3). Figures 3 and 4 are showing the course of effect on bloating and abdominal discomfort over time, exemplarily. The placebo effects were only measurable after 4 weeks of intake, whereas the Perilla extract effects were measurable already in the first part of intervention and were even strengthened with ongoing supplementation.

Additional descriptive calculation of responder rates indicated in Perilla group higher responder rates in comparison to placebo (Perilla: bloating 83%, passage of gas 67%, GI rumbling 71%, feeling of fullness 67%, abdominal discomfort 71% // Placebo: bloating 57%, passage of gas 52%, GI rumbling 57%, feeling of fullness 52%, abdominal discomfort 48%). This was especially pronounced for “bloating” with 83% responder in Perilla versus 57% in placebo group.

**Figure 5:**
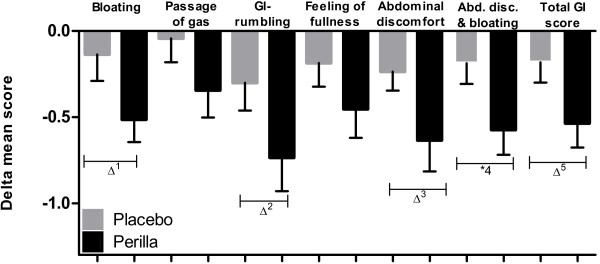
**Mean differences of GI symptoms.** GI symptoms between the last two weeks of supplementation and the run-in phase for women. (∆^1^: p = 0.0691; ∆^2^: p = 0.0951, ∆^3^: p = 0.0676; *^4:^ p = 0.048 ∆^5:^: p = 0.0621 , unpaired t-test).

In the subgroup of women, the reduction of GI symptoms by supplementation of Perilla extract appeared even more pronounced. All five GI symptoms decreased significantly over time within the perilla group (repeated measures ANOVA) (bloating: -0.51 ± 0.57, p = 0.0002; passage of gas: -0.34 ± 0.60, p = 0.0272; GI rumbling: -0.74 ± 0.85, p = 0.0002; feeling of fullness: -0.45 ± 0.73, p = 0.0062; abdominal discomfort: -0.63 ± 0.79, p = 0.0004), whereas in the placebo group none of the symptoms decreased significantly over time (see also Table 4).The benefit of perilla in comparison to placebo in the subgroup of women was seen by trend for the following GI symptoms: bloating (95%CI -0.031 to 0.79, p = 0.069) (Figure 3), GI rumbling (95%CI -0.080 to 0.95, p = 0.095) and abdominal discomfort (95%CI -0.0314 to 0.83, p = 0.068) (Figure 4). In women, the summary score out of all 5 GI symptoms showed significant reduction in the Perilla group (-0.54 ± 0.61, p = 0.0001). The symptoms decreased from 1.37 ± 0.71 at run-in, over 1.11 ± 0.54 after 2 weeks of intake to 0.83 ± 0.56 after 4 weeks of intake. This reduction of GI symptoms in the Perilla group showed a difference by trend in comparison to placebo (95%CI -0.02 to 0.73, p = 0.062) (Figure 5). The comparison of the sub-score including bloating and abdominal discomfort showed statistically significant results between Perilla and placebo in the female group (95%CI 0.003 to 0.77, p = 0.048) (Figure 5).

**Table 4:** Mean values and standard deviation (SD) for women of Gastrointestinal symptoms and the delta changes between the run in phase and the intake phase (week 3-4)

**Women (N = 38)**	**Placebo**				**Perilla**				**Placebo**	**Perilla**	
	**Run In**	**Intake week 1-2**	**Intake week 3-4**	**p value (ANOVA)**	**Run In**	**Intake week 1-2**	**Intake week 3-4**	**p value (ANOVA)**	**Delta week 3-4 to run in**	**Delta week 3-4 to run in**	**Comparison between groups (95% CI)**
Bloating	1.54	1.65	1.4	0.1641	1.43	1.27	0.91	0.0002	-0.14	-0.51	-0.031 to 0.787
SD	0.67	0.58	0.68		0.85	0.71	0.83		0.67	0.57	
Passage of gas	1.54	1.69	1.49	0.2572	1.62	1.51	1.28	0.0272	-0.04	-0.34	-0.126 to 0.727
SD	0.65	0.47	0.59		0.81	0.68	0.77		0.60	0.69	
GI rumbling	1.6	1.47	1.3	0.1685	1.64	.1.17	0.9	0.0002	-0.30	-0.74	-0.080 to 0.948
SD	0.78	0.92	0.85		0.81	0.59	0.68		0.70	0.85	
Feeling of fullness	1.05	1.13	0.86	0.1016	0.96	0.76	0.51	0.0062	-0.19	-0.45	-0.173 to 0.709
SD	0.78	0.61	0.67		0.79	0.67	0.59		0.60	0.73	
Abdominal discomfort	1.14	1.16	0.91	0.0843	1.2	0.83	0.57	0.0004	-0.24	-0.63	-0.031 to 0.828
SD	0.87	0.84	0.84		0.89	0.64	0.65		0.48	0.79	

For the comparison within group p values of repeated measures ANOVA and for the comparison between groups the 95% confidence intervals of differences are shown.

The descriptive responder analysis in women also showed higher responder rates for the Perilla extract group in comparison to placebo. (Perilla: bloating 84%, passage of gas 74%, GI rumbling 79%, feeling of fullness 68%, abdominal discomfort 74% // Placebo: bloating 47%, passage of gas 47%, GI rumbling 58%, feeling of fullness 47%, abdominal discomfort 47%).

These effects were most pronounced for “bloating” with 84% responders in Perilla versus 47% in Placebo group.

The delta changes of stool frequency between the run-in and the intervention phase did not show significant differences between groups. In the placebo group number of days with stool per week increased about 0.8 ± 1.02 and slightly higher about 0.83 ± 1.4 in the Perilla group. Stool consistency assessed with Bristol Stool Form Scale tended to become softer in both groups. The placebo group improved from 2.42 ± 0.86 to 3.2 ± 1.1 and Perilla from 2.47 ± 1.01 to 2.9 ± 1.1. The total score of PAC-SYM was significantly improved in both groups (Placebo: 95%CI 0.246 to 0.699, p = 0.0002; Perilla: 95%CI 0.152 to 0.520, p = 0.0257). However, in comparison between groups there were no significant differences. Overall reported symptoms and changes in symptoms were very low and in most cases only around the category 1 (mild). The results of the PAC-QOL showed also no significant changes between groups. The results of the Perceived Stress Questionnaire (PSQ20) showed no differences within and between groups during intervention.

Most of subjects followed the order not to change their nutrition or sports habits during the study. Only two subjects reported about somewhat more and one subject about somewhat less physical activity. Nevertheless these deviations were only minor and no interference with study results is expected.

At each visit, adverse events and concomitant medication were documented. Most adverse events were headache and common colds, which were not related to the study product. One subject was down with gastroenteritis. A relationship to the study product is improbable.

## Discussion

The results of the human study show that Perilla extract is able to improve gastrointestinal discomfort symptoms. Perilla extract improved all GI discomfort symptoms (bloating, passage of gas, GI rumbling, feeling of fullness and abdominal discomfort) significantly over the 4-week intervention. Placebo was only able to reduce abdominal discomfort significantly after 4 weeks of intake. The placebo effects were only measurable after 4 weeks of intake, whereas the Perilla extract effects were measurable already in the first part of intervention and were even strengthened with ongoing supplementation. Perilla extract showed consistently higher reduction in gastrointestinal symptoms compared to placebo. In female subjects, these differences were even more pronounced than in the whole study group, and bloating, GI rumbling as well as abdominal discomfort were improved by trend in the Perilla extract group if compared to placebo. Furthermore, the sub-score including bloating and abdominal discomfort showed significant results for the female group (p = 0.048). Especially in females, but also in the whole study population, the Perilla extract group showed consistently higher responder rates to gastrointestinal symptom relief. Responder rates demonstrated physiological relevance of Perilla extract for reduction of GI symptoms. The results of the trial confirm that Perilla extract is able to improve the postulated main targeted GI discomfort parameter, referring to its mode of action, as Perilla extract has proven prokinetic and anti-spasmodic efficacy, beneficial to reduce these symptoms [7,21]. In the study the effects of Perilla extract in comparison to Tapioca starch, a fully digestible carbohydrate (placebo control), on gastrointestinal discomfort and bowel function were investigated. The study was conducted according to a double-blind and placebo-controlled design with a 4-week intervention period. Gastrointestinal discomfort often accompanied by bloating or rumbling, is a common symptom in otherwise healthy adults. The aim of the study was the evaluation of Perilla extract on a representative group of the general population. Therefore a population with reduced bowel movement and gastrointestinal discomfort, but otherwise healthy was included. Approximately 20% of the population, particularly women, suffers from GI discomfort and these disorders affect quality of life [1,2]. To meet the higher number of affected women in the general population, more women than men were included. The challenge of the study was, to demonstrate improvements of GI symptoms in the general population with only little occurrence, especially as available evaluation tools in most cases are developed for a diseased population [13,14]. Therefore one reason for performing a pilot trial with Perilla extract, besides the containment of the area of gut health where Perilla extract shows its potential, was to find suitable tools for the healthy population with only little occurrence of symptoms. The daily documentation of GI symptoms including bloating, passage of gas, GI rumbling, feeling of fullness and GI discomfort, was a direct measure of occurrence of a set of predominant gastrointestinal symptoms which was very well accepted by participants. Nevertheless, the sensitivity of the questions could be increased in further studies by increasing the rating scale, to possibly meet the little occurrence of symptoms. The problematic of a lack of recognized markers for measuring gastrointestinal well-being and digestive symptoms in the general population was also assessed by Guyinnet et al. [22]. Possibly, this newly developed tool as a measure of GI well-being improvement can also be added in further studies. As symptoms of gastrointestinal discomfort and the constipation of stool are more or less intravariable acts, it is important to have a run-in period, long enough, to have meaningful baseline values. In the current study, baseline conditions for GI discomfort and bowel function parameters assessed during the run-in phase were comparable between groups and volunteers suffered at least 6 months from reduced bowel movements. The data of the perceived stress questionnaire confirmed, that there was no change of overall stress level during the study. Also no relevant changes of nutrition or activity status during the study were documented.

However, there were some limitations of the study. Until the beginning of the study, there was no experience from literature, in which area of gastrointestinal discomfort Perilla extract shows primary effectiveness. However, due to *ex vivo* and *in vi*tro results a balancing effect was anticipated. Therefore a lot of parameters concerning gastrointestinal discomfort were assessed during the study and no primary objective was defined, thus the character of a pilot study was chosen. Due to lack of literature with Perilla extract in the field of gut health or GI symptoms, no a priori sample size calculation was performed and determination of sample size was only estimated according to literature with similar questions. The results show very promising results, and it seems as Perilla extract is able to improve gastrointestinal symptoms in the observed population. Nevertheless, the results could not demonstrate significant advantage of Perilla extract over placebo for each parameter even if there are clear hints. An underestimated sample size or less sensitivity of used questionnaires has to be considered as possible reasons for lacking significance. Results are also offering indication for future studies, which tools are suitable and which are potential primary objectives for evaluation of Perilla extract’s effects in the area of digestive health. On this basis, further research with higher sample size and possibly adapted questionnaires will confirm the effectiveness of Perilla extract to reduce gastrointestinal discomfort.

Another challenge of research in the field of GI discomfort is the known placebo effect for human studies investigating gastrointestinal health [10,11]. Literature reports indicate that placebo effects in studies on gastrointestinal function range from 10% to 70% for functional dyspepsia [23] and 0% to 84% for irritable bowel syndrome [11]. In the current study also a high placebo effect could be observed in each parameter (around 60%), which complicates the recognition of clear effects. Nevertheless the results of the study show an advantage for Perilla extract over placebo. Consumer research indicates that today one third of consumers looking for gut health support do not find an effective product that alleviates any of their discomfort. In addition also patients with irritable bowel syndrom or irritable bowel disease wish to apply or as a matter of fact use food supplements or complementary and alternative medicines [24,25]. Many indications for the use of such remedies are anecdotally or traditionally derived. Physicians are looking for alternative therapies, co- medications or food supplements which demonstrated beneficial effects within randomized controlled trials. The promising results shown for Perilla extract, a food grade ingredient, could benefit consumer with GI discomfort and patients suffering from GI diseases.

## Conclusion

The demonstrated effects of Perilla extract to improve GI discomfort offer very promising results taking into consideration the challenging set up of a nutritional human study with healthy volunteers and in the area of digestive health, which is known for high placebo effects.

### Ethical approval

Ethical approval was obtained from the ethical committee of the “Landesärztekammer Baden-Württemberg” prior to study start.

## Abbreviations

ANOVA: Analysis of variance; BMI: Body mass index; ICH-GCP: International conference on harmonisation of technical requirements for registration of pharmaceuticals for human use – guideline for good clinical practice; GI symptoms: Gastrointestinal symptoms; PAC-SYM: Patient assessment of constipation symptoms; PAC-QOL: Patient assessment of constipation quality of life; PSQ20: Perceived stress questionnaire; SD: Standard deviation; 95% CI: 95% confidence interval.

## Competing interests

The study was sponsored by Amino Up Chemical Co., Ltd. and Vital Solutions GmbH. The sponsors contributed to discussion about study design and selection of outcome measures prior to study start. During study realization and data analysis all data were completely blinded and study realization, data analysis and report generating was undertaken independently from the sponsor. Amino Up Chemical Co. Ltd. and Vital Solutions GmbH own the proprietary ingredient used in the study by equal shares.

## Authors’ contributions

The study was designed by CAR and CS (Biotesys GmbH) after approach from SBW (Vital Solutions GmbH) and HF (Amino up Chemical Co., Ltd.). CAR and CS undertook management of the study, including study execution, overseeing data collection, management, quality assurance and analyses. All authors contributed to the study design and data interpretation. CAR and CS wrote the first draft of this paper and all authors were responsible for subsequent critical revision of the manuscript. SBW. is the corresponding author for this paper. All authors read and approved the final manuscript.

## Authors’ information

Christiane Schön (Dipl. Nutrition science) and Claudia Reule (PhD Sports science and examin. biol.), both clinical research scientists at BioTeSys GmbH, a company with over 10 years experience in the field of nutrition research and clinical nutrition studies. BioTeSys GmbH, Schelztorstrasse 54-56, Esslingen, D-73728, Germany.

Hajime Fujii, Chemist, PhD, over 20 years experience in the natural product industry for health and nutrition, President/ COO and head of R&D, at Amino Up Chemical Co., Ltd., 363-32 Shin-ei, Kiyota, Saporo, Japan.

Sybille Buchwald-Werner, Pharmacist, PhD in Pharmaceutical Chemistry, over 14 years experience in the natural product industry for health and nutrition. Managing Director and head of R&D at Vital Solutions GmbH, Hausingerstrasse 6, Langenfeld, 40764, Germany.

## Pre-publication history

The pre-publication history for this paper can be accessed here:

http://www.biomedcentral.com/1472-6882/14/173/prepub
